# A KNOWLEDGE-DRIVEN BIO-BEHAVIORAL NETWORK MODEL OF THE INFLAMMASOME AND CALORIC RESTRICTION

**DOI:** 10.1093/geroni/igad104.3140

**Published:** 2023-12-21

**Authors:** Thomas Chacko, Gordon Broderick, Nadia Moore, Daniel Parker, William Kraus, Kim Huffman

**Affiliations:** Rochester Regional Health, Rochester, New York, United States; Rochester Regional Health, Rochester, New York, United States; Rochester Regional Health, Rochester, New York, United States; Duke University School of Medicine, Durham, North Carolina, United States; Duke University, Durham, North Carolina, United States; Duke University, Durham, North Carolina, United States

## Abstract

Healthy dynamic equilibrium between immune and endocrine function can be altered not only by illness but by changes in lifestyle including caloric intake. The Comprehensive Assessment of the Long-term Effects of Reducing Intake of Energy Study (CALERIE™) is a large multi-year longitudinal study funded by the National Institute on Aging investigating what metabolic adaptations would result from a 25% caloric restriction sustained for 2 years and how these might positively affect primary aging and inform on aging biomarkers and chronic diseases of aging. A broad range of behavioral and physiological markers were measured at different levels of biology, making comprehensive integration and interpretation of data challenging. Here we propose a knowledge-based framework for integrating data that fully applies our current understanding of biobehavioral regulatory biology. Leveraging the Elsevier Knowledge Graph and the MedScan Natural Language Processing (NLP) engine we extracted from peer-reviewed literature 816 documented regulatory relationships linking a subset of 85 data elements readily available, including ~ 15 immune inflammatory and 12 endocrine markers. With a connection density of ~11%, this network was supported by > 23,000 citations or a median of 7 citations per regulatory interaction. A structural analysis of connectivity patterns identified inflammatory marker CCL2, insulin and testosterone as key mediators of large-scale coordinated response to caloric restriction. Ongoing analysis will identify immune and endocrine decisional logic that drives network dynamics, providing candidate intervention targets potentiating caloric restriction that remain explainable in the context of established pathway biology.

